# Supplementary material for: Hospital unit working conditions and risk for employee injury
Source: PLoS One. 2025 Dec 18;20(12):e0339151. doi: 10.1371/journal.pone.0339151 (PMC12714235; doi:10.1371/journal.pone.0339151)
Supplement: S1 File — This single Supporting Information file contains all supplementary materials associated with the manuscript, including: Table S1 (characteristics of employees and injury events under passive surveillance), Table S2 (sociodemographic and occupational profile of the active-surveillance cohort), Table S3 (mixed-effects logistic regression results without active-surveillance predictors), Table S4 (multivariable results excluding injuries reported in the past week), and Table S5 (mixed-effects logistic regression including active-surveillance predictors). (DOCX) [file pone.0339151.s001.docx]

**Hospital Unit Working Conditions and Risk for Employee Injury**

**Online Supplement**

Emrah Gecili^a,b^, Nancy M. Daraiseh^a,b^, Cole Brokamp^a,b^, Maurizio Macaluso^a,b^

## **Table S1**. Characteristics of employees and injury events recorded under passive surveillance. This is for the employees in the units evaluated during the period in which the passive surveillance data were collected.

| **Characteristic** | **Category / Statistic** | **n (%) or Summary** |
| --- | --- | --- |
| Number of employees | — | 1214 |
| Gender of injured staff | Female | 1,024 (84) |
|  | Male | 190 (16) |
| Age at incident (years) | Mean (SD) | 33.3 (9.9) |
|  | Range | 19–66 |
| Most frequent job categories | Registered Nurse | 617 (51) |
|  | Mental Health Specialist I / II | 237 (20) |
|  | Patient Care Assistant | 122 (10) |
|  | Other clinical / support roles | 238 (19) |
| Primary nature of injury | Pain / Strain | 296 (24.4) |
|  | Abrasion / Laceration | 191 (15.7) |
|  | No symptoms reported | 144 (11.9) |
|  | Puncture wound | 91 (7.5) |
|  | Bruise | 54 (4.4) |
| Principal cause of injury | Patient – aggressive behavior | 541 (44.6) |
|  | Patient – non-aggressive | 85 (7) |
|  | Self / lack of situational awareness | 81 (6.7) |
|  | PPE not used / failure | 77 (6.3) |
|  | Work environment issues | 40 (3.3) |
| Inpatient units represented | — | 25 |

## **Table S2.** Sociodemographic and occupational profile of the active-surveillance cohort (N = 607)

| **Characteristic** | **Category** | **n (%)** |
| --- | --- | --- |
| Gender | Female | 522 (86) |
|  | Male | 75 (12) |
|  | Missing | 10 (2) |
| Age group (years) | <25 | 144 (24) |
|  | 25–29 | 189 (31) |
|  | 30–34 | 106 (17) |
|  | 35–44 | 90 (15) |
|  | ≥45 | 61 (10) |
| Race / ethnicity | Non-Hispanic White | 493 (81) |
|  | Non-Hispanic Black | 76 (13) |
|  | Other racial/ethnic groups | 23 (4) |
| Current position | Registered nurse | 437 (72) |
|  | Mental health specialist | 81 (13) |
|  | Patient care attendant | 89 (15) |
| Shift schedule | 8-hour shifts | 187 (31) |
|  | 12-hour shifts | 242 (40) |
|  | Combination (8 & 12 h) | 177 (29) |
|  | Other schedules | 1 (<1) |
| Unit of employment | Medical–surgical | 447 (74) |
|  | Psychiatry | 160 (26) |

**Table S3.** Risk factors influencing presence of employee injury on a given day. Results from mixed effects logistic regression without considering active surveillance predictors (N=7929).

|  | **Single-predictor model** | | **Multivariable model** | |
| --- | --- | --- | --- | --- |
| **Predictor** | OR (crude) (95% CI) | p-value | aOR (95% CI) | p-value |
| % shifts > 13 h (for one additional % point): |  |  |  |  |
| on the same day | 1.02 (0.99,1.05) | 0.2336 | 1.00 (0.96, 1.05) | 0.8238 |
| 1 day before | 1.03 (1.01, 1.06) | 0.0096* | 1.04 (1.01, 1.07) | 0.0118* |
| 2 days before | 1.01 (0.98, 1.04) | 0.6362 | 1.00 (0.97, 1.03) | 0.9086 |
| 3 days before | 1.01 (0.98, 1.04) | 0.6164 | 1.01 (0.98, 1.04) | 0.5885 |
| N days in the past week when %shifts > 13 h increased over the previous day (per additional day) | 1.01 (0.92, 1.11) | 0.7983 | 0.92 (0.83, 1.02) | 0.1295 |
| % shifts < 8.5 h on the same day (for one additional % point) | 0.99 (0.98, 0.997) | 0.0056* | 0.99 (0.97, 1.02) | 0.6111 |
| % shifts between 8.5 & 12 h in a unit on the same day (for one additional % point) | 1.01 (1.002, 1.02) | 0.0148* | 1.00 (0.97, 1.03) | 0.9218 |
| % of overtime h in the past week (for one additional % point) | 1.02 (0.95, 1.10) | 0.5579 | 1.00 (0.92 1.08) | 0.9909 |
| N days in the past week when the %ADC decreased from the previous day (per additional day) | 1.05 (0.96, 1.16) | 0.2614 | 1.07 (0.97, 1.17) | 0.1720 |
| N days in the past week when the % of nurse orientation hours tr decreased from the previous day | 0.93 (0.86, 1.001) | 0.0525 | 0.95 (0.88, 1.02) | 0.1701 |
| N injuries reported in the past week (per additional injury reported) | 1.19 (1.12, 1.27) | <0.0001* | 1.15 (1.07, 1.24) | 0.0003* |
| Any exposure to bodily fluids (no BBP exposure) in the past week (yes/no) | 1.47 (0.81, 2.64) | 0.2029 | 1.29 (0.72, 2.33) | 0.3954 |
| Aggressive patient Injury (yes/no) | 1.57 (1.24, 1.98) | 0.0002* | 1.20 (0.91, 1.58) | 0.1954 |

*: significant at 0.05; **: significant at 0.1; BBP: Blood-borne pathogen; N: number; OR: odds ratio; aOR: adjusted odds ratio; CI: confidence interval.

**Table S4.** Risk factors influencing the presence of employee injury on a given day. Results from mixed effects logistic regression without considering active surveillance predictors (N=7929). The difference from Table S1 is excluding the number injuries reported in the past week from the model to investigate potential over adjustment.

|  | **Multivariate model** | |
| --- | --- | --- |
| **Predictor** | **aOR (95% CI)** | **p-value** |
| % shifts > 13 h (for one additional % point): |  |  |
| on the same day | 1.01 (0.97, 1.05) | 0.7265 |
| 1 day before | 1.04 (1.01, 1.07) | 0.0093* |
| 2 days before | 1.01 (0.97, 1.03) | 0.8265 |
| 3 days before | 1.01 (0.98, 1.04) | 0.5029 |
| N days in the past week when %shifts > 13 h increased over the previous day (per additional day) | 0.93 (0.84, 1.04) | 0.2070 |
| % shifts < 8.5 h on the same day (for one additional % point) | 0.99 (0.96, 1.02) | 0.6055 |
| % shifts between 8.5 & 12 h in a unit on the same day (for one additional % point) | 1.00 (0.97, 1.03) | 0.8631 |
| % of overtime h in the past week (for one additional % point) | 1.01 (0.93, 1.09) | 0.8531 |
| N days in the past week when the %ADC decreased from the previous day (per additional day) | 1.05 (0.96, 1.16) | 0.2598 |
| N days in the past week when the % nurse orientation hours decreased from the previous day | 0.93 (0.86, 1.00) | 0.0534** |
| Any exposure to bodily fluids (no BBP exposure) in the past week (yes/no) | 1.45 (0.80, 2.61) | 0.2168 |
| Aggressive patient Injury (yes/no) | 1.53 (1.21, 1.95) | 0.0004* |

*: significant at 0.05; **: significant at 0.1; BBP: Blood-borne pathogen; N: number; OR: odds ratio: aOR: adjusted odds ratio; CI: confidence interval.

**Table S5.** Risk factors Influencing Presence of Employee Injury on a Given Day.

Results of Mixed Effects Logistic Regression Including Active Surveillance Predictors (N=1517)

|  | **Univariate model** | | **Multivariate model** | |
| --- | --- | --- | --- | --- |
| **Predictor** | **OR (crude) (95% CI)** | **p-value** | **aOR (95% CI)** | **p-value** |
| % shifts > 13 h (for one additional % point): |  |  |  |  |
| on the same day | 1.03 (0.97, 1.09) | 0.3406 | 1.03 (0.95, 1.11) | 0.5055 |
| 1 day before | 1.01 (0.95, 1.08) | 0.7657 | 0.98 (0.92, 1.06) | 0.6781 |
| 2 days before | 1.02 (0.96, 1.09) | 0.5334 | 1.00 (0.93, 1.07) | 0.9928 |
| 3 days before | 0.97 (0.90, 1.05) | 0.4937 | 0.94 (0.87, 1.03) | 0.1820 |
| N days in the past week when %shifts > 13 h increased over the previous day (per additional day) | 1.13 (0.95, 1.35) | 0.1715 | 1.19 (0.97, 1.47) | 0.0967** |
| % shifts < 8.5 h on the same day (for one additional % point) | 1.01 (0.99, 1.02) | 0.216 | 1.01 (0.96, 1.05) | 0.8029 |
| % shifts between 8.5 & 12 h in a unit on the same day (for one additional % point) | 0.99 (0.98, 1.00) | 0.1740 | 1.00 (0.95, 1.05) | 0.9419 |
| % of overtime h in the past week (for one additional % point) | 1.00 (0.87, 1.14) | 0.9960 | 0.99 (0.87, 1.12) | 0.8153 |
| N days in the past week when the %ADC decreased from the previous day (per additional day) | 0.88 (0.73, 1.07) | 0.1990 | 0.93 (0.77, 1.13) | 0.4601 |
| N days in the past week when the % nurse orientation hours decreased from the previous day | 0.81 (0.69, 0.94) | 0.0069* | 0.88 (0.76, 1.02) | 0.0805** |
| N injuries reported in the past week (per additional injury reported) | 1.37 (1.17, 1.60) | <0.0001* | 1.30 (1.05, 1.62) | 0.0176* |
| Any exposure to bodily fluids (no BBP exposure) in the past week (yes/no) | 1.96 (0.74, 5.18) | 0.1727 | 1.28 (0.45, 3.58) | 0.6444 |
| Aggressive patient Injury (yes/no) | 1.07 (0.67, 1.71) | 0.7718 | 0.70 (0.41, 1.22) | 0.2120 |
| N near-misses reported in the past week (per additional injury reported) | 1.15 (1.04, 1.27) | 0.0049* | 1.10 (0.98, 1.23) | 0.1125 |

*: significant at 0.05; **: significant at 0.1; BBP: Blood-borne pathogen; N: number; OR: odds ratio: aOR: adjusted odds ratio; CI: confidence interval.
