# Supplementary material for: Hospital unit working conditions and risk for employee injury
Source: PLoS One. 2025 Dec 18;20(12):e0339151. doi: 10.1371/journal.pone.0339151 (PMC12714235; doi:10.1371/journal.pone.0339151)
Supplement: Attachment — Submitted filename: Response to Reviewers-Pt-by-pt revisions PONE-D-25-05110.docx [file pone.0339151.s002.docx]

**File: Point-by-point responses to reviewers**

Manuscript ID: **PONE-D-25-05110**

***Denotes authors’ point-by-point responses

*We sincerely thank the Associate Editor and Reviewers for their thoughtful assessment and feedback, which have helped improve our manuscript. Below, we provide our responses and refer to the tracked changes version, where additions and deletions are marked accordingly. Below, our responses are indicated with *** and presented in italics.*

**Reviewer #1:**

**R1.1**: Ambiguities in the Methods Section:
*Data Collection Period and Scope:
- The study mentions two different data collection periods:
-- The period for collecting employee safety reports (2014–2017)
-- The participant sampling and data collection period (2013–2015)
The rationale for this discrepancy is not explained. It remains unclear whether these different data collection processes are directly comparable.
**Recommendation: The integration of these datasets should be clarified to ensure consistency and comparability.

****Authors’ response: Thank you for your comment. The decision to integrate both passive and active injury surveillance datasets was intentional and central to our study’s objectives. Passive surveillance, while efficient and broad in scope, often underreports injuries due to its reliance on existing records and reporting mechanisms. In contrast, active surveillance involves proactive data collection and tends to capture a more accurate picture of injury occurrences. Merging these datasets allows for a more comprehensive evaluation of employee injuries. This clarification has been added to section 2.1 Data Collection.*

**R1.2**: Insufficient Definition of Key Variables:
*Lack of a Clear Definition for Patient Aggression:
- Although patient aggression is a key variable in the statistical analysis, its definition (e.g., physical assault, verbal threats, etc.) is not clearly stated.
**Recommendation: A precise definition of patient aggression should be provided to ensure clarity and reproducibility of the findings.

****Authors’ response: Thank you for your comment. Indeed, this concern is related to a similar comment by Reviewer 6. Please see comment* ***R6.7*** *and our response below. Patient aggression was defined per institution policy as any aggressive/violent behaviors to others and verbal aggression/threats/agitation. This has been added to section 2.1 Data Collection.*

**R1.3**: Potential Over-adjustment in Statistical Models:
*Collinearity Between Independent Variables:
- The significance of patient aggression disappears when previous injuries are included in the same model, which may indicate an over-adjustment issue, potentially masking the true effect.
**Recommendation: Alternative modeling approaches should be considered to examine the independent effects of these variables. For instance, separate models could be tested for patient aggression and previous injuries to assess their individual contributions.

****Authors’ response: We thank the reviewer for this important observation. We agree that the diminished significance of the patient aggression variable when controlling for prior injuries may reflect over-adjustment due to collinearity between the two predictors. To address this, we conducted additional analyses to assess their individual and joint contributions to injury risk.* ***Assessment of Collinearity:*** *We examined the Pearson correlation between the number of injuries in the past week and patient aggression, which was r = 0.66, indicating a strong positive relationship. To more formally assess collinearity in the regression models, we also inspected the covariance/correlation matrix of parameter estimates and calculated variance inflation factors (VIFs). VIF values for most predictors, including patient aggression (VIF = 1.32) and number of injuries (VIF = 1.39), were low, suggesting that collinearity between these two predictors was modest. Overall, these results indicate that while multicollinearity between patient aggression and injury counts is present, it is not severe; nonetheless, their conceptual and statistical overlap may contribute to attenuation of the aggression effect when both are included in the same model.*

**Alternative Modeling Approaches:** To assess the potential for over-adjustment, we estimated three models:

- **Model 1:** Includes only aggressive patient injury as a predictor. In this model, aggressive patient injury was a significant predictor (OR = 1.53, 95% CI: 1.21–1.95).
- **Model 2:** Includes both aggressive patient injury and number of injuries in the past week. In this model, the effect of aggressive patient injury is no longer statistically significant (OR = 1.20, 95% CI: 0.91–1.58), while past week injuries remain significant (OR = 1.15, 95% CI: 1.07–1.24).
- **Model 3:** Includes only number of injuries in the past week. In this model, past injuries remain a robust predictor of injury risk (OR = 1.18, 95% CI: 1.10–1.26).

These results support the reviewer’s concern: when both predictors are included, the effect of aggressive patient injury is attenuated, likely due to overlap with recent injury history. Although VIF values for both predictors were low (~1.3–1.4), their strong correlation (r = 0.66) and conceptual relatedness suggest over-adjustment. Alternative model comparisons showed that aggression alone was a significant predictor, past injuries alone were also robust, but when combined, past injuries dominated and the aggression effect lost significance. Importantly, we now also acknowledge that increased frequency of injuries in a unit may itself elevate the risk of subsequent aggressive patient behavior, placing aggression on the causal pathway between a high-risk environment and employee injury. In this case, the two variables cannot be considered fully independent covariates, and future work using mediation analysis will be needed to disentangle direct and indirect effects.

**Conclusion:** We have revised the manuscript to note that patient aggression and past injury counts are not only correlated but may also be causally linked. We clarify that each predictor independently associates with injury risk, but joint inclusion likely masks the aggression effect because of this causal overlap. Additional explanation has been added to the Results and Discussion to highlight this possibility and point toward mediation analysis as a more appropriate framework for future work. *Please see page 15 and lines 321-334 and page 19 and lines 413-423.*

**R1.4**: Sample Size and Generalizability Concerns:
*Single-hospital Sample:
- The study is based on data from a single pediatric hospital, which may limit the generalizability of the findings to other healthcare settings.
**Recommendation: The study’s limitations should be explicitly stated, and the need for future multi-center studies should be emphasized to improve external validity.
****Authors’ response: We appreciate the reviewer's observation regarding the limitation of our study being conducted at a single pediatric hospital, which may affect the generalizability of our findings. We acknowledge that single-center studies can limit external validity due to factors such as unique institutional practices, patient demographics, and staffing models. To address this concern, we edited the* ***Discussion*** *and* ***Conclusions*** *sections. Please see page 21 and lines 449-453 and page 25 and lines 540-543.*

**R1.5**: Limitations in Data Sharing:
*Restricted Data Access:
- The statement "The data that support the findings of this study are confidential and not publicly available." may raise concerns about the transparency and reproducibility of the study.
**Recommendation: If possible, summary statistics or anonymized datasets should be made available to enhance transparency and facilitate replication of the findings.

****Authors’ response: Thank you for your comment. We appreciate the reviewer's concern regarding the transparency and reproducibility of our study, particularly in relation to data availability. We acknowledge the importance of providing access to data that support research findings. However, due to the sensitive nature of the data involved in our study, which includes identifiable employee information and confidential institutional records, we are unable to make the full dataset publicly available. To address this concern, we have revised our data availability statement.*

**Reviewer #2:**

Unfortunately, I cannot recommend publication of the article due to several major shortcomings in both its design and its relevance and methodological rigor.

****Authors’ response: We respectfully disagree with the reviewer's assessment. We have tried to address the specific comments and hope that the revised manuscript is acceptable for publication.*

**R2.1**: Temporal relevance of the data: Data collection in the study was conducted 10 years ago. This temporality presents a significant limitation since the results and conclusions of the study may have lost relevance in the current nursing context. Health and nursing research is constantly evolving, and old data may not reflect current conditions, needs, and practices in the discipline. This weakens the external validity of the study and its applicability to contemporary practice.
****Authors’ response: We thank the reviewer for this important observation. We fully acknowledge that the temporal distance from data collection to publication represents a limitation that could influence the generalizability and external validity of our findings. However, we respectfully argue that the core structural and organizational risk factors examined—such as extended shifts, staffing shortages, nurse orientation levels, and exposure to patient aggression—remain persistent challenges in healthcare, including pediatric inpatient settings. The mechanisms linking these workplace conditions to injury risk continue to be highly relevant, even as specific contextual features of clinical care evolve.*

*Moreover, the insights from this historical data provide a baseline perspective that can inform understanding of how such risk factors operated before the transformative disruptions introduced by the COVID-19 pandemic. We have revised the* ***Discussion*** *section to explicitly address this temporal limitation and position the findings as both historically informative and foundational for comparison with more recent studies. Please see pages 23-24 and lines 498-507.*

**R2.2**: Few references and lack of updating: The article presents only 18 bibliographic references, and the most recent ones date from 2020. This limited number of references and the lack of updating in the bibliographic sources evidence a deficiency in the integration of the most recent advances in the scientific literature on the subject. This calls into question the completeness of the theoretical framework and the state of the art of the study, which is crucial to ensure that the research is well grounded in the most recent developments in the discipline.

****Authors’ response: In response, we have conducted a comprehensive review of the most recent scientific literature published between 2021 and 2025 that is relevant to occupational injuries in healthcare settings, fatigue and shift work, and injury prevention.*

*We identified several recent studies and reviews that have reinforced the importance of factors such as extended shifts, rotating schedules, and cumulative workload in relation to workplace injury risk among healthcare workers.*

*We have revised the* ***Introduction****,* ***Discussion, References*** *sections to incorporate these recent studies.*

**R2.3**: Insufficiently detailed methodology: Although the study mentions that it uses mixed methods, no clear explanation of how the qualitative part of the research was conducted is provided. Nor is the qualitative part stated in the results.
****Authors’ response:* *We thank the reviewer for this observation. We would like to clarify that although our study involved the use of voice recordings, which were qualitative in form, the objective was not to conduct a qualitative thematic analysis or a traditional mixed methods study as typically defined in health services research (i.e., combining qualitative and quantitative research questions, methods, and analyses). Instead, we used the voice-recorded reports to generate structured, coded variables (e.g., occurrence of near-misses) for inclusion in our quantitative modeling. To avoid confusion, we note that the term “mixed models” in the manuscript refers to regression models with both fixed and random effects, not to mixed methods research.*

**R2.4**: Lack of information on the number of participants: The methodology does not specify the number of participants involved in the study. This omission is a serious problem since the number of participants is an essential factor in assessing the validity and reliability of the results. Without this information, it is impossible to judge the representativeness of the sample or the statistical power of the analyses, which directly affects the robustness of the findings.
****Authors’ response:* *We thank the reviewer for this important observation. We have clarified the study populations and analytic units for both data sources.
The passive surveillance dataset included 7,929 unit-days of aggregated surveillance and operational data collected across 25 inpatient units, encompassing approximately 1,214 employees from intensive care, medical–surgical, and psychiatry services.
The active surveillance dataset contributed 1,517 unit-days derived from 607 employees who participated in a time-limited observational study where staff recorded injuries and near-misses using voice recorders (see Table S1 and Table S2). Because analyses were conducted at the unit-day level, the associations detected represent relationships between unit-level exposures and injury risk rather than individual-level effects. We have clarified these sample sizes and aggregation levels in both the Methods (Data Sources) and Results (Opening paragraph) sections for transparency.*

**R2.5**: Absence of reference to EQUATOR guidelines: The article does not explicitly mention which good practice guidelines and recommendations, such as those of EQUATOR, were followed during the design and execution of the study. The EQUATOR guidelines are fundamental to ensure that research is conducted following international ethical and methodological standards. The omission of this information suggests that the study may not have adhered to best practices in terms of transparency and methodological rigor.
Given the combination of these factors, I believe that the manuscript does not meet the minimum requirements of quality and relevance necessary to be published in a scientific nursing journal. I suggest that major revisions be made in terms of methodology, updating of references, and temporal relevance of the data before it is considered for publication.

****Authors’ response: We appreciate the reviewer’s emphasis on best practice guidelines. We would like to clarify that not explicitly stating adherence to such standards in the original manuscript does not imply that they were not followed. This study was designed and reported in accordance with international best practice guidelines, specifically* ***STROBE (Strengthening the Reporting of Observational Studies in Epidemiology)****, which is the appropriate EQUATOR guideline for observational studies. We have now updated the Methods section to explicitly state this adherence and ensured that all relevant checklist items are addressed in the manuscript.*

*We also recognize the importance of temporal context and have added text clarifying the impact of the COVID-19 pandemic and changes in healthcare delivery since the data collection period. In addition, we have updated our reference list to reflect recent literature and methodological developments.*

**Reviewer #3:**

This study investigates risk factors for employee injuries in a pediatric hospital setting through analysis of self-reported injury data, active surveillance via voice recording, and hospital unit measures. Using mixed effects logistic regression models, the authors identified several significant risk factors, including shifts exceeding 13 hours, patient aggression, and prior injury occurrences.
This research addresses a critical real-world issue with significant implications. Identifying risk factors for hospital worker injuries is essential for improving healthcare workplace environments and employee wellbeing. However, there are several points author need to consider:

**R3.1**: The authors have not provided essential descriptive information about the sample population. Details regarding the participants' gender, age, education level, job roles, and work experience would provide important context for interpreting the results and understanding the generalizability of the findings.

****Authors’ response: We agree and have expanded the descriptive information for both datasets. The passive surveillance sample is organized at the unit-day level and therefore does not include individual-level demographic data. However, Table S1 now summarizes employee-level characteristics aggregated from available records, showing that most staff were female (84%) and employed in direct-care roles such as registered nurses (51%) and mental-health specialists (20%).*

*For the active surveillance cohort, detailed sociodemographic and occupational information—including age distribution, race/ethnicity, and shift schedule—is provided in Table S2, adapted from Macaluso et al. (2018, Scand J Work Environ Health). In brief, the majority of participants were female (86%), white (81%), younger than 30 years (55%), and employed as registered nurses (72%), with a median of two years in their current role.*

*Together, these descriptions establish the context and representativeness of the hospital workforce captured in our analytic models. We have added reference to these details in the Results to provide demographic context and now acknowledge in the Discussion that the absence of individual-level demographic data for the passive surveillance dataset is a limitation, while emphasizing that it nonetheless reflects a large and diverse hospital workforce across multiple inpatient units.*

**R3.2**: While the authors mention that their findings can "guide the development of targeted interventions," the manuscript would benefit from a more detailed discussion of specific strategies hospitals could implement based on these findings. For example, how might hospitals modify shift scheduling policies or address patient aggression more effectively?
****Authors’ response: We thank the reviewer for this insightful suggestion. In response, we have expanded the* ***Discussion*** *section to provide a more detailed and evidence-based overview of actionable interventions that hospitals can implement based on our findings. Specifically, we discuss strategies related to shift scheduling—such as limiting extended shifts, predictive scheduling, and scheduled rest breaks—and approaches to managing patient aggression, including de-escalation training, early identification of high-risk patients, response teams, and feedback-based incident reporting systems. We believe these additions provide practical guidance for healthcare administrators and contribute to the development of safer working environments.*

**R3.3**: The study appears to rely on pre-pandemic data, which limits its contemporary relevance. Given the significant impact of COVID-19 on healthcare work environments, the authors should include a discussion of how the pandemic may have altered risk factors and working conditions for healthcare employees, and how their findings might translate to the post-pandemic context.
****Authors’ response: We thank the reviewer for raising this important point. We acknowledge that our study was conducted using pre-pandemic data, which may not fully reflect the shifts in healthcare environments brought about by COVID-19. In response, we have added a dedicated paragraph in the* ***Discussion*** *section addressing the potential impact of the pandemic on healthcare work conditions, risk factors for injury, and how our findings might be interpreted in the post-pandemic context. Specifically, we discuss how the pandemic has likely intensified some of the risk factors identified in our study—such as extended shifts, staffing shortages, and patient aggression—due to increased workload, emotional strain, and ongoing staffing challenges. We also acknowledge the emergence of new risk factors such as PPE-related physical stress, burnout, and infection control demands. However, we argue that the core findings of our study remain relevant, as the fundamental mechanisms—fatigue, workload, and environmental stressors—continue to influence injury risk. We also highlight the need for future research using post-pandemic data to validate and expand upon these results. Please see* ***pages 22-23 and lines 481-497.***

**R3.4**: The authors should provide more comprehensive information about their data processing methods, particularly regarding how they coded and analyzed the voice recordings from active surveillance. This additional methodological transparency would strengthen the validity and reproducibility of the research.

****Authors’ response: The methodology for transcript coding and quantification is detailed in a separate publication Macaluso et al. 2018 (14), which we reference in the manuscript. For full transparency and reproducibility, we encourage readers to consult that publication for a comprehensive description of the procedures used.*

**Reviewer #4:**

Thank you for the opportunity to review this work.
With this, I have a few questions:
**R4.1**: For methods, I am curious as to the nature of the dataset in general (given that it isn't included in the supplemental materials). Typically for a study of this nature, the dataset itself is usually described (e.g. 'x' number of survey responses, with 'y' being excluded for 'z' reason, resulting in 'c' subjects left for analysis...). I would also indicate that this is a retrospective review of prospectively collected data (which is something I am assuming per line 167 in the manuscript). As such, there are usually variables that are missing in such data, and I would be interested to know if that resulted in subjects / responses being excluded.

****Authors’ response: We thank the reviewer for highlighting the need for greater clarity about the dataset structure, exclusions, and missing data handling. In response, we revised both the* ***Methods*** *and* ***Results*** *sections to address these points. In the* ***Methods****, we now explicitly describe the study as a retrospective analysis of prospectively collected institutional data, and explain that listwise deletion was used to handle missing data at the unit-day level. In the* ***Results****, we added the initial sample sizes for both analytic datasets (with and without active surveillance predictors), the number of unit-day records excluded due to missing data, and the final sample sizes used in the analysis. This addition provides greater transparency regarding data processing and sample flow. Please see page 10 and lines 222-227 and pages 12 and lines 258-259.*

**R4.2**: In any case, I presume that the total population of survey results you are analyzing is 9446 (7929 without active surveillance predictors and 1517 with, per Table 1). My confusion stems from whether or not the with/without active surveillance predictor datasets are supposed to represent a case/control type analysis. And I presume the intent was to compare data from when the employees were encouraged to include near-misses in reporting, though the manuscript doesn't make clear what the exact difference between "with active surveillance" and "without active surveillance" means in terms of the study population.
****Authors’ response: We thank the reviewer for this thoughtful comment and the opportunity to clarify our study design. To address the concern:*

*First, we confirm that this is not a case-control study. The datasets “with” and “without” active surveillance predictors represent different unit-day analytic samples, not different groups of individuals. The dataset without active surveillance includes all inpatient unit-day records for which passive surveillance and operational data were available. The dataset with active surveillance is a subset of that data, restricted to unit-days from a time-limited observational study where employees on selected units were asked to record injuries and near-misses via voice recorders.*

*The active surveillance dataset does not represent a separate “treatment” group or period. Instead, it enhances data quality and completeness for a subset of unit-days, particularly around near-miss events and aggressive patient encounters. The intent was not to compare these two datasets as case and control groups, but to explore whether including active surveillance-derived predictors adds explanatory value to the models.*

*We note that a formal comparison of active and passive surveillance systems has already been reported (Macaluso et al., 2018 (14)). In this manuscript, our focus is on modeling injury risk, not re-comparing surveillance modalities. To clarify this in the manuscript, we have revised the* ***Methods (Data Collection)*** *section to describe the origin, purpose, and usage of the active surveillance data. We also added language explicitly stating that the two datasets are not meant to be directly compared in a case-control framework. Please see page 11 and lines 238-240.*

**R4.3**: Along with this, there does not appear to be any evaluation as to time-of-day an injury might occur. Correlating injury to time-of-shift may be helpful, as other papers have noted that "flex time" shifts (evening followed by daytime, for example) are a potential risk factor for injury. Given changes in circadian rhythm, this makes intuitive sense, but is not addressed in this paper.
****Authors’ response: We thank the reviewer for this observation. We agree that injury timing—including time-of-day and time within a shift—is a critical factor in understanding injury risk and has been highlighted in previous literature on circadian rhythm disruption and shift transitions. Our current analysis was limited to unit-day level data, and we did not have access to more granular data. To maintain the scope of this paper, we restricted our analysis to unit-day predictors, but we agree that time-of-day and shift-pattern analyses represent a valuable next step. We plan to address these questions in subsequent work.*

*In response to this comment, we revised the* ***Discussion*** *to acknowledge this limitation of scope and to highlight time-of-day and shift-pattern analysis as a key direction for future research. Please see page 22 and lines 466-475.*

**R4.4**: In addition, although you show a forest plot with an indication that there is a significant (but small) odds ratio increase for longer shifts, there does not seem to be a dose-response to such ratios - which seems counter-intuitive if fatigue is the mechanism by which these injuries are thought to occur. Most notably, the percentage increase of over 13-hour shifts the same day did NOT show a corresponding statistically significant increase in reported injuries, similar to the lack of change with the percent (number?) shifts over 13h in past week. (I am not clear as to if the "N days in past week with % shifts > 13 h increased" refers to the percentage of shifts over 13 hours in a week, or the number of over-13 hour days in a given week as the variable).
****Authors’ response: We appreciate the reviewer's insightful observations and the opportunity to clarify the variables related to shift lengths exceeding 13 hours.*

***Clarification of Variables: i) Percentage of Shifts >13 Hours (Same Day):*** *the proportion of shifts exceeding 13 hours on a given day within a unit.* ***ii) Number of Days in Past Week with % Shifts >13 Hours Increased:*** *number of days in the preceding week during which the percentage of shifts exceeding 13 hours was higher than that of the previous day. It captures the frequency of days with an increasing trend in extended shifts, reflecting potential periods of escalating workload or staffing shortages.*

***Interpretation of Findings:*** *Our analysis identified associations between certain shift length variables and injury risk; however, a clear dose-response relationship was not consistently observed. Specifically: The percentage of shifts exceeding 13 hours on the same day did not show a statistically significant association with reported injuries. Similarly, the number of days in the past week with an increased percentage of shifts over 13 hours did not exhibit a significant relationship with injury occurrences.*

*We* *emphasize that our analyses are conducted at the unit-day level. As such, associations we detect likely represent a dampened reflection of the underlying individual-level relationships.* *Several factors may further explain the absence of a dose-response pattern: 1) Fatigue Mitigation Strategies: Units may implement measures such as scheduled breaks, task rotation, or additional staffing during longer shifts to counteract fatigue, thereby reducing the impact of extended work hours on injury risk.; 2) Adaptation and Resilience: Staff members accustomed to longer shifts may develop adaptive strategies or resilience, mitigating the effects of fatigue associated with extended work hours. 3) Underreporting Bias: There is a possibility that injuries occurring during extended shifts are underreported, possibly due to increased workload, time constraints, or normalization of fatigue-related symptoms among staff.*

*While some studies have reported a dose-response relationship between shift length and injury risk, findings across the literature are mixed. For example, the National Institute for Occupational Safety and Health (NIOSH) reports a 28% increase in accident and error risks associated with 12-hour shifts compared to 8-hour shifts, suggesting a potential dose-response effect. However, variations in study designs, populations, and operational contexts can lead to differing results. To further elucidate the relationship between shift length and injury risk, future studies should consider: 1) gathering detailed information on individual shift patterns, exact shift durations, and corresponding injury timestamps to enable precise analysis.; 2) Incorporating objective measures of fatigue and alertness to directly assess their mediating role between shift length and injury occurrences. Please see page 9 and lines 200-203* *and page 19 and lines 396-400.*

**R4.5**: As it stands, the data you are presenting suggest that scheduling 13-hour shifts increase injuries, while scheduling <8.5 hour shifts can reduce injuries - though that notion intuitively doesn't help when trying to schedule a provider for, as an example, three 13-hour shifts per week vs 5 or 6 8.5-hour shifts per week, as the at-risk work time exposure per individual worker is different.
****Authors’ response: We appreciate the reviewer’s careful consideration. Our results show that on days with more long shifts (>13 hours), injury risk is higher, while on days with more short shifts (<8.5 hours), injury risk is lower. Importantly, our analysis was conducted at the shift-day level and did not account for the number of workers at risk. Thus, the observed associations may in fact be conservative: when more employees work shorter shifts, the denominator of workers at risk increases, yet the overall injury risk remains lower.*

*We also note that the reviewer’s scenario of “equal total weekly work hours” does not fully capture cumulative fatigue and recovery time. Multiple consecutive long shifts are well documented to impair recovery and elevate risk, independent of the total hours worked. Our findings are consistent with this broader occupational health literature, which highlights increased risk associated with long shifts. The persistence of 12–13 hour scheduling in practice often reflects worker preference, rather than evidence of reduced risk. Please see page 18 and lines 388-395.*

**R4.6**: On that note, I do wonder how your regression analysis was set up. Reviewing the tables, it's evident that the number of shifts is entered as a percentage variable, while the number of "days in past week" for three variables are set as an integer ("n"), along with injuries reported and near misses reported. Was there a reason to use "percentage" over integer values when noting numbers of shift lengths? If integer values were used, the conclusion would theoretically read "for every individual shift worked by unit staff over 13h there is an increased 'x' risk for injury", which is easier to understand. Conversely, a tighter association with fatigue might be present if "hours worked by staff 7 days prior" substituted for the shift variables - or at least "number of >13h shifts worked by unit staff in 7 days prior". I would argue that such dependent variables would be better, as they are not
****Authors’ response: In our regression models, In our analysis, the predictors were constructed to reflect the relative distribution of shift lengths within each unit, which is why percentages were used for shifts rather than absolute counts. This standardization enabled meaningful comparisons across units of varying sizes. We note, however, that the risk factors are not directly interpretable at the level of individual shifts because our models are based on unit-level aggregated data rather than individual worker-shift exposures. A full per-shift or per-hour interpretation would require denominators capturing the number of staff at risk per day, which were not available in our dataset. We have clarified this in the Methods section and added text in the Discussion to acknowledge that future work incorporating more granular denominator data would allow for more precise interpretations of injury risk at the individual level. Please see pages 9-10 and lines 211-221.*

**R4.7**: I also note that your manuscript lists "percent NHPPD" and "Unit RN Vacancy" as potential risk factors. Those variables are not visibly noted in your regression. Is there a reason why these were left off?
****Authors’ response:* *We thank the reviewer for catching this inconsistency. The mention of “percent NHPPD” and “Unit RN Vacancy” was carried over from an earlier draft and should have been removed, as these variables were not included in our final regression analyses. We apologize for the oversight and have corrected the manuscript accordingly.*

**R4.8**: I would also be interested to know how the data analysis was performed (e.g. SASS, STATA?). Your data collection and database analysis method isn't listed in the manuscript. Also, the use of a mixed-effect logistic regression was described, however the statistical analysis used in the univariate calculation of "injury from patient aggression" (line 251-252) was not described.
****Authors’ response: Thank you for your comment. We appreciate the reviewer’s request for clarification. All statistical analyses were performed in R (version XX.X, R Foundation for Statistical Computing, Vienna, Austria) using the lme4 package for mixed-effects logistic regression and base R functions for descriptive and univariate analyses. The univariate association of injury from patient aggression was assessed using simple logistic regression models. We have updated the Methods section (please see subsection “2.2 Statistical Analysis”) to include this information and to explicitly describe the software, packages, and statistical approaches used for both multivariable and univariate analyses.*

**R4.9**: Finally, the issue of relevance to healthcare administrators with regards to this paper comes into question. Namely, is there data regarding the severity of the injuries encountered? One can argue that administrators would be more concerned about the long-term risk for injury, as it would be different between a potential blood-borne contact with no long-term sequelae vs an assault that resulted in worker-days lost. Given the nature of this analysis, my supposition is that the number of injuries sustained that were severe enough to result in a lost day at work were not enough to analyze properly - though I do wonder if the reason "injury reported in past week" variable was significant because it reflected a continuation of an injury sustained by a staff member the week prior.
****Authors’ response: Thanks for the comment. Our dataset did capture OSHA‐recordable events (i.e., injuries resulting in death, days away from work, restricted duty, transfer, or medical treatment beyond first aid), and these are noted in the manuscript. However, such events were relatively rare during the study period, limiting the statistical power to analyze them separately.*

*For this reason, and to capture the broader safety climate within hospital units, our primary analyses focused on all reported injuries, regardless of severity. This approach is consistent with institutional safety surveillance practices, where both minor and serious events contribute to understanding patterns of risk and prevention opportunities. The significant association observed with “injuries reported in the past week” likely reflects an overall high‐risk environment rather than being driven exclusively by recurrence of severe injuries.*

*We now clarify this in the manuscript and emphasize in the Discussion that future research with larger samples or pooled multi‐institutional data could stratify by severity to better inform administrators about the impact of different types of injuries (e.g., those leading to lost workdays vs. minor events). To address these considerations, we added a paragraph to the* ***Discussion*** *section. Please see page 22 and lines 461-466.*

**R4.10**: As an aside, most trauma and injury prevention literature already utilize "ISS" as an abbreviation for "injury severity score". As this paper does not list the severity of injuries, rather than document the self-reported existence or absence of such, I'd suggest that your abbreviation should be changed to "IISS", for "institutional injury surveillance system", in order to avoid confusion.
****Authors’ response: Thank you for suggestion regarding the potential for confusion between the commonly used abbreviation "ISS" for "Injury Severity Score" and our use of "ISS" for "Institutional Injury Surveillance System". To prevent any ambiguity and align with standard terminology in trauma and injury prevention literature, we have updated our manuscript to use the abbreviation "IISS" for "Institutional Injury Surveillance System".*

**R4.11**: As for formatting, including a legend embedded within your figures (vs having them within the body of the manuscript - see lines 234-240, for example) would be helpful. And is there a reason that your table of model results are only within the supplemental material, rather than as a table within the main manuscript?

****Authors’ response: Thank you for these suggestions aimed at enhancing clarity. To ensure consistency with the journal's formatting requirements, we have retained the figure legends within the main text. Since Figures 1 and 2 already provide all the information presented in the supplemental tables (model results), we included them in the supplement.*

**Reviewer #5:**

A great article !
I have some reservations
**R5.1**: Regarding the discussion, it needs to be more elaborate and please mention study populations. As this study dealt with pediatric.
Several factors differ.
****Authors’ response: We appreciate the reviewer's insightful feedback. To address these concerns, we have expanded the Discussion section to provide a more comprehensive analysis of our findings, particularly in the context of the pediatric population. We have explicitly highlighted how the unique characteristics of pediatric healthcare settings and patient demographics may influence employee injury risks.*

**R5.2**: As mentioned you have active and passive surveillance factors, I would want a bit of explanation on this regard.
****Authors’ response: Thank you for your comment regarding clarification of active and passive surveillance. In our study, passive surveillance refers to the institutional 803-SAFE call-in system, through which staff report injury events, while active surveillance refers to the use of handheld digital recorders by a sample of staff to document injuries and near-misses during their shifts. This distinction has been clarified in the revised Methods section. For additional methodological details on the active surveillance component, please see Macaluso et al. 2018 (14), which provides the full study design and procedures.*

**R5.3**: on 224 line “unadjusted analyses”), a 1% = Bracket just appeared closed end, while there was no open ended.
****Authors’ response: We have corrected the unmatched closing bracket to ensure clarity and proper punctuation.*

**R5.4**: Table 1 needs more explanation

****Authors’ response: We appreciate the reviewer's observation regarding Table 1. To enhance its clarity and utility for readers, we have slightly revised the table, its caption, and footnote. Additionally, we have edited and extended the paragraph explaining Table 1 under Results section.*

**Reviewer #6:**

This manuscript reports a study that aims to explore and quantify the risk factors associated with employee injuries on inpatient units. It appears that an additional aim was to explore the value of adding an active approach to incident surveillance – that of carrying a pocket voice recorder to more proactively capture near misses.
The study analyzed employee injury data self-reported to the institutional surveillance system, as well as incident reports collected from a sample of employees through active surveillance (voice recording), and hospital unit-based measures of patient density and employee workload as potential determinants of injury
The main findings were that shifts of >13 hours on the prior day and patient aggression are positively associated with injury likelihood and shifts <8.5 hours on the same day are negatively associated.
Overall, the work reported covers an important topic, reports new information from the pediatric setting, and appears to be a well conducted study.
**R6.1**: Some sections of writing are too lengthy, omit important information, or are in the wrong section. Referencing is too thin throughout, particularly in the Introduction. Attention is also needed in the Discussion. Suggestions for improving the clarity and organisation of writing are given below.
****Authors’ response: We have revised the manuscript and references to improve clarity and organization.*

**R6.2**: Table 1 is not easy to follow and needs review of how information is presented to make clear what it is, and clean up a few inconsistencies. Statistical review is recommended.

****Authors’ response: Thanks for the feedback. We acknowledge the importance of presenting data in a clear and consistent manner. In response to the reviewer's comment, we have revised Table 1 to improve its clarity and consistency. This included providing clear and descriptive title, consistent formatting of entries, defining all abbreviations, presenting appropriate statistical measures, and including explanatory footnotes as needed. These measures aim to enhance the readability and interpretability of the tables presented in this manuscript.*

**R6.3**: References are too thin throughout. All statements of fact need to be substantiated with evidence. An introduction of the different types of incident surveillance system data collection methods (e.g., online, call centre, pocket recorder) etc is needed given the exploration of this aspect in the study.
****Authors’ response: We thank the reviewer for the suggestion. We have updated the Introduction and Methods sections to strengthen referencing throughout and to explicitly introduce the main types of incident surveillance approaches used in healthcare settings, including online systems, call-center reporting, and active surveillance methods such as digital recorders. We have also cited key studies (e.g., Macaluso et al., 2018 (14)) and related institutional reporting literature) to support these statements and improve clarity on how our study fits within the broader context of injury surveillance research.*

**R6.4**: Lines 102-119 are excessively wordy. Any justification for the study needs to be presented earlier in the section. Any postulation about the meaning and impact of the study needs to go in the Discussion. These paragraphs should be reduced to max 1-2 short sentences that state the study aim(s). Details of the methods should go in the Methods section.
****Authors’ response: We thank the reviewer for highlighting the need for a more concise and focused Introduction. In response, we have revised lines 102–119 to succinctly state the study's objectives and have relocated detailed methodological descriptions to the* ***Methods*** *section. Please see page 5 on lines 112-117.*

**R6.5**: Start by stating the study design. Identify a study reporting guideline appropriate to the study design, review your manuscript against this and attach the check list as an appendix, and note which guideline you used in the first paragraph of the methods.
****Authors’ response: We appreciate the reviewer's suggestion to clearly state the study design and adhere to an appropriate reporting guideline. To address this, we have revised the* ***Methods*** *section of our manuscript to explicitly describe the study design and confirm adherence to the relevant reporting standards.*

**R6.6**: Make clear that the study is conducted in a single hospital. (Mentioned in the aims at end of previous section – suggest move to Methods).
****Authors’ response: We thank the reviewer for highlighting the need to specify the study setting more explicitly. In response, we have revised the* ***Methods*** *section to clearly state that the study was conducted at a single, large Midwestern pediatric hospital. This clarification ensures that readers are aware of the study's context and the potential implications for generalizability. Please see page 5 and lines 124-126.*

**R6.7**: Clearer introduction of inclusion/exclusion criteria is needed. Definitions of some of the variables is needed. For example, how is ‘patient aggression’ defined and measured?
****Authors’ response: We acknowledge the importance of clearly delineating the inclusion and exclusion criteria to enhance the reproducibility and transparency of our study. Additionally, we recognize the necessity of providing precise definitions for key variables, such as "patient aggression," to ensure consistent interpretation. Please see page 6 and lines 133-135.*

**R6.8**: Regarding data collection, clearer reporting is needed of how data were extracted, from what systems in what format, by whom.
****Authors’ response: We revised the Methods (specifically, Section 2.1) to clarify how data were extracted and prepared for analysis. Specifically, we note that IISS reports were exported from the hospital’s electronic reporting platform in structured text format by the occupational safety team, while active surveillance recordings were transcribed and coded by trained research staff before integration with the IISS dataset.*

**R6.9**: Line 167-76: There appears to be a staff population subgroup with separate method of data collection for a short period within the study timeframe. Is this the ‘Active surveillance’ data? Suggest give this a sub-heading. Introduce and justify. Please briefly describe the larger study from which this group was drawn and how potential participants for this study were identified and by whom they were invited. Clarify how this data is analysed.
****Authors’ response: We thank the reviewer for raising this point. To clarify, the active surveillance data did not represent a distinct staff subgroup but rather a complementary dataset that we integrated with the institutional injury surveillance system (IISS). The rationale for integration is that active surveillance captures near misses—events that are typically underreported in passive systems—making it critical for understanding the broader risk of injury. Random samples of RNs, PCAs, and BHSs in Med/Surg and psychiatry units were invited to participate in the active surveillance study, and consenting participants (N = 607) recorded injury events and near misses using handheld voice recorders for a two-week period. These recordings were transcribed, coded into structured variables, and incorporated into our quantitative models rather than analyzed thematically.*

*We have revised the* ***Methods*** *section (Data Collection) to make this clearer, emphasizing that the active surveillance data complemented rather than constituted a separate subgroup, and that its integration provided richer information on underreported precursors such as near misses.*

**R6.10**: It would be helpful if the total number of staff, hours worked, and number of units from which data was drawn could be reported in the opening of the Results section.
****Authors’ response: We appreciate the reviewer’s suggestion to clarify the organizational scope of the study. As noted in our response to Reviewer 2, the passive surveillance dataset comprised 7,929 unit-days of aggregated surveillance and operational data collected across 25 inpatient units, while the active surveillance dataset contributed 1,517 unit-days derived from 607 employees who recorded injuries and near-misses using voice recorders.*

*We have revised the opening of the Results section to include these details and to explicitly state that the analytic unit is the unit-day, representing all staff scheduled to work on a given unit during a given day. These revisions clarify both the temporal and organizational scope of the datasets and provide appropriate context for interpreting the aggregated model results.*

**R6.11**: Line 210-4: Judgement, opinion and other interpretation should be reserved for the discussion. Recommend remove from here ‘Notably’ and full sentence of Lines 212-4.
****Authors’ response: We appreciate the reviewer's observation regarding the placement of interpretive language in our manuscript. We revised the manuscript accordingly and removed Lines 212-4.*

**R6.12**: Table 1: Some of the values are presented in variable format and it is not clear what the % represent – i.e., of what total. Please review for clarity and completeness.
****Authors’ response: We appreciate the reviewer's attention to the clarity of percentage representations in Table 1. To address this concern and enhance the comprehensibility of our data presentation, we have undertaken a thorough review and revision of Table 1, ensuring that all percentage values are clearly defined and contextualized.*

**R6.13**: Lines 305-8: A suggestion is for this sentence to form the beginning of the next paragraph
****Authors’ response: We appreciate your input and have repositioned the sentence to the start of the following paragraph.*

**R6.14**: Line 323: Needs referencing

****Authors’ response: We appreciate the reviewer's suggestion. Recent studies across various industries have reinforced the critical role of near-miss reporting in enhancing workplace safety, and we have added multiple references to support this.*

**Reviewer #7:**

I commend the authors for their thorough work investigating the risk factors associated with hospital employee injuries. The authors effectively contextualized the present issue and described the need for this research. Clear descriptions of hospital injury procedures, variables of interest, and data analysis methodology are presented. The authors captured near-misses along with recorded injuries, which provides information not typically investigated when studying workplace safety. Potential over-adjustment of the statistical model and collinearity between variables were adequately addressed. Tables and figures provide are clear and provided all relevant data. Overall results demonstrate the importance of employee shift duration and prompt responses to injuries for employee safety. Based on their findings, the authors suggested several areas requiring improvements to healthcare management and administration to enhance employee safety.

A few minor suggestions are provided below to enhance clarity.
****Authors’ response: We sincerely thank the reviewer for their thorough and thoughtful evaluation of our manuscript. We are pleased that the contextualization of the issue, the clarity of our methodology, and the inclusion of near-miss data were well-received. Below, we have carefully addressed the minor suggestions provided to enhance clarity.*

**R7.1**: For clarity regarding the five metrics assessing risk factors for employees, the authors are recommended to provide more precise relationships between the measure and what the measure indicates. This was done for metrics 1, 2, and 3, however, readers may benefit from clearer descriptions for metrics 4 and 5. For metric 4, the authors are suggested to specify the value that may indicate decreased skill level or experience. For metric 5, the authors are suggested to specify that higher values may be an indicator of staff fatigue. These changes will improve clarity for readers.
****Authors’ response: Thank you for your feedback. We agree that clarifying the interpretations of metrics 4 and 5 will enhance the manuscript's clarity. Metric 4 (Nurse Orientation Hours):
We have specified that a value exceeding 15% may indicate a decreased skill level or experience among staff, potentially impacting patient care and safety. Metric 5 (Shifts ≥13 Hours): We have clarified that higher percentages of shifts lasting 13 hours or more may be indicative of staff fatigue, which can increase the risk of errors and injuries. Please see page 7 and lines 155-161.*

**R7.2**: In the results sections, the authors are recommended to specify variable units of measurement to clarify values for the reader. Although clearly described in the methods section, there is limited mention that measures were captured at the unit level. For example, in line 263–264, the authors could include that the number of near-misses were recorded for each unit (e.g., “The average number of near-misses reported in the past week was 0.65 (SD 1.53) per unit in the active surveillance dataset”). This addition would clarify the results for readers who may skip the methods section.
****Authors’ response: We appreciate the suggestion to enhance clarity regarding the units of measurement in the Results section. To address this, we have revised the manuscript to explicitly state that the reported measures were captured at the unit level. Specifically, we have updated the sentence on lines 263–264.*

**R7.3**: Table 1 has some inconsistent formatting and unnecessary characters that can be removed.
a. Percentages are not included in the table consistently across variables, which may confuse readers. For example, the categorical variable, Injury Occurrence, includes a percentage sign within the parentheses to denote that 477 is 6.02% of the 7929 participants included in the study. However, % shift > 13 h provides continuous data, but the use of a percent sign within the parentheses impedes reader understanding of whether the values within the parentheses for this variable are SDs for a mean or percentages for n cases. With percentages and Ns specified in the variables column and the table footnote providing additional information, the authors are recommended to remove the percentages from the table columns containing data.
b. Similarly, the use of “days” in the data columns (e.g., for the variable N days in the past week when % shifts >13 h increased over the previous day) is not necessary and can be removed as it adds noise to the table. The “days” qualifier has already been stated in the Variables column, which provides sufficient information for each variable.
c. The authors are recommended to write out the ADC acronym in the footnote.
These changes would improve readability as well as consistency with the supplemental table.
Overall, the authors provided novel data regarding hospital employee injury risk factors. Results were adequately presented and the study findings are adequately interpreted. Author concerns and study limitations were appropriately addressed throughout the manuscript and yielded opportunities to discuss the complexity of this field of research.

****Authors’ response: We thank the reviewer for this helpful suggestion. Based on the feedback, we have revised Table 1 to ensure consistent formatting and clearer presentation. Specifically, we removed unnecessary percentage signs from the data columns, retained percentages only where appropriate for categorical variables, and clarified Ns and percentages in the variable column and table footnote. For continuous variables, we now report means (SD) without percent signs to avoid confusion.*
