# Supplementary material for: Hospital unit working conditions and risk for employee injury
Source: PLoS One. 2025 Dec 18;20(12):e0339151. doi: 10.1371/journal.pone.0339151 (PMC12714235; doi:10.1371/journal.pone.0339151)
Supplement: Attachment — Submitted filename: R2- Response to Reviewers-Pt-by-pt PONE-D-25-05110.docx [file pone.0339151.s003.docx]

**File: Point-by-point responses to reviewers**

Manuscript ID: **PONE-D-25-05110**

***Denotes authors’ point-by-point responses

*We sincerely thank the Associate Editor and Reviewers for their thoughtful assessment and feedback, which have helped improve our manuscript. Below, we provide our responses and refer to the tracked changes version, where additions and deletions are marked accordingly. Below, our responses are indicated with *** and presented in italics.*

**Reviewer #3:** The authors have adequately addressed the concerns raised in the previous round of review. The revisions have improved the clarity and rigor of the manuscript. I have no further comments at this stage.

****Authors’ response: We thank Reviewer #3 for the positive evaluation and appreciate the acknowledgement of our revisions. We are glad the updates improved the clarity and rigor of the manuscript.*

**Reviewer #4**: Thank you for your responses to my concerns.
I would note that for the response re: R4.5 - "Thus, the observed associations may in fact be conservative: when more employees work shorter shifts, the denominator of workers at risk increases, yet the overall injury risk remains lower."
I'd argue that the converse is true - if the standard work-week is 40 hours, on a 10-RN unit (for example), an 8-hour shift system would require 42 FTE's to staff the 210 slots fully for one week, without overtime.
Conversely, a "39-for-40" system with 13-hour shifts (presuming half-hour overlap at each end) would require 47 nurses to cover the same unit over 140 slots, if there's no overtime. So there are more FTE's in the 12-hour-shift scenario needed vs the 8-hour.

****Authors’ response: We thank the reviewer for this helpful clarification. We agree that, from a scheduling perspective, different shift-length systems (e.g., 8-hour vs. >13-hour extended shifts) require different full-time-equivalent (FTE) staffing configurations. As the reviewer illustrates, a schedule built on >13-hour shifts may require more individual staff than an 8-hour system to ensure full weekly coverage. We have revised the Discussion to reflect this important point and to clarify how it relates to our analytic approach.*

*At the same time, our analytic unit was the unit-day—not the individual worker—so we could not directly model the number of staff physically present or the underlying FTE structure associated with each scheduling system. This means our results reflect unit-level associations between the distribution of shift lengths and injury risk, rather than per-worker risk.*

*Importantly, even if >13-hour systems sometimes require more staff overall, the workers assigned to these extended shifts experience substantially greater fatigue and reduced recovery time than those working shorter shifts. This aligns with extensive evidence showing that extended daily hours—particularly >12–13 hours—are associated with elevated injury risk due to cumulative fatigue, sleep debt, and decreased vigilance. We have revised the Discussion to incorporate these clarifications.* ***Please see pages 18-19 and lines 389-399.***
